# Navigating the Forest of Lignin-Derived Monomers for Polymer Synthesis

**DOI:** 10.1021/acs.biomac.6c00494

**Published:** 2026-06-30

**Authors:** Peter Olsén, Elena Subbotina

**Affiliations:** † Wallenberg Wood Science Center, Laboratory of Organic Electronics, Linköping University, Norrköping 60174, Sweden; ‡ Laboratory of Organic Electronics, 7655 Linköping University, Norrköping 60174, Sweden

## Abstract

The field of lignin-based polymeric materials is undergoing rapid development, driven by increasing sustainability demands. However, progress in lignin-derived materials is often pursued from different disciplinary perspectives—biomass chemistry, organic synthesis, and polymer materials science—using field-specific metrics, resulting in fragmented knowledge. This Perspective examines the lignin-to-materials pathway by connecting advances in the conversion of lignin into platform molecules, their transformation into monomers, and the synthesis of polymeric materials through representative examples. We perform rough estimates of sustainably available lignin streams and compare them with current polymer production, indicating that lignin could potentially supply aromatic monomers at scales comparable to existing markets. Through analysis of key literature on lignin-to-monomers and monomers-to-polymer strategies, we identify critical directions for lignin-to-materials development. These include refinery concepts that utilize complex lignin-derived substrates as primary building blocks, prioritizing the use of their inherent functionality before stepwise defunctionalization, and adopting application-driven materials design, in which the requirements of a target application guide monomer and polymer selection rather than attempting to reproduce the molecular structures of the petroleum-derived polymers currently used for those applications.

## Graphical abstract

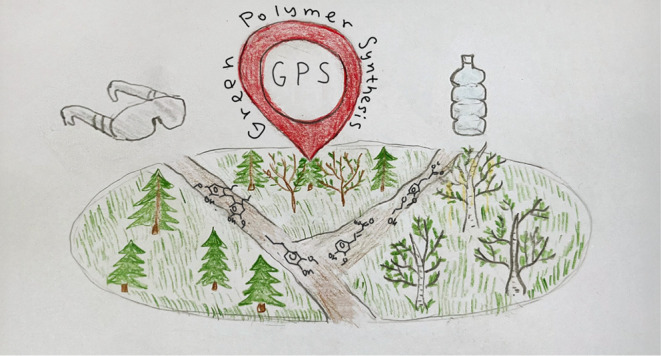

## Introduction

This perspective aims to critically discuss the current state of the art and the future potential of lignocellulosic biomass—specifically lignin—as a source of monomers for polymer synthesis. The scope is deliberately limited to pathways involving deconstruction of lignin into well-defined molecular building blocks, followed by their chemical modification into polymerizable monomers. Pathways in which lignin is used directly in its macromolecular form are therefore excluded and are discussed elsewhere.^1–5^

Transition toward renewable feedstocks for the chemical industry is a central objective of sustainable development and is explicitly reflected in several United Nations Sustainable Development Goals (SDGs). In this context, lignocellulosic biomass represents the largest practically accessible renewable carbon resource. Within lignocellulose, lignin stands out as the largest natural source of renewable aromatic compounds. The total global demand for aromatics (benzene, toluene, xylene (BTX) fraction) in the chemical industry is estimated at approximately 130–150 Mt yr−1 ^6^ (*Mordor Intelligence*), mainly used for the production of polymeric materials, including commodity plastics, thermosets, adhesives, paints, and coatings. This illustrates the strategic importance and explains the research interest in lignin-to-polymers valorization as a renewable alternative to fossil-derived aromatic materials.

This perspective is motivated by the need to summarize and critically assess the current state of the art and future prospects of lignin-to-materials pathways, and in particular to bring together two research areas that often emphasize different aspects of the same value chain: (i) fractionation and conversion of lignin into well-defined low molecular weight products, and (ii) the synthesis of polymeric materials from renewable aromatic building blocks. Although major advances have been made in both areas, they are frequently pursued with different primary objectives and evaluation criteria, resulting in limited alignment in terms of process realizability, the scale of potentially available monomer streams relative to polymer market volumes, and performance metrics relevant for polymer applications.

## Lignocellulose Availability

Lignocellulose is the principal structural component of plant biomass and is found in wood, grasses, and agricultural residues such as sugarcane bagasse, corn cobs, and wheat straw. Global lignocellulosic biomass generation has been estimated at ∼181 Gt yr^–1^, while current utilization is ∼8–9 Gt yr^–1^.^7^ To assess the feasibility of lignin-to-materials pathways, we derive order-of-magnitude estimates of sustainably available lignocellulosic resources.

To estimate sustainably available biomass by 2050, several modeling approaches have been proposed. Here, we primarily rely on a recent study from LUT University that used the novel LUT-Bioenergy model to provide estimates at high geospatial and temporal resolution.^8^ The model considers forestry, agriculture, and household wastes, of which forestry and agricultural biomass are the most relevant here due to their well-characterized composition and suitability for lignin valorization. The model evaluated two scenarios: Business-as-Usual (BAU) and Toward Sustainability (TSS). The BAU scenario extrapolates current production and consumption trends, whereas TSS reflects transitions toward more sustainable food systems and reduced environmental pressure.^9–11^ The values reported in this perspective represent the average of the BAU and TSS scenarios. Sustainably available forestry lignocellulose comprises residues from logging and mechanical wood processing (e.g., sawmilling, plywood production), excluding black liquor from chemical pulping. Agricultural residues include crop residues and livestock manure as defined in the LUT framework; inedible abattoir residues are excluded.

Based on this work and assuming an average lignocellulose energy content of 16 MJ kg^–1^, sustainably available lignocellulosic biomass from forestry and agriculture by 2050 is estimated at ∼1.1 and ∼3.9 Gt yr^–1^, respectively (Figure 1A). These values are consistent in order of magnitude with other global biomass potential assessments, despite methodological differences.^12,13^ Worth mentioning, that a substantial fraction of current forestry removals is still used as fuelwood. Considering this, the future feedstock availability could further increase if woody biomass shifts from low-grade energy use toward higher-value material and chemical applications.^14,15^

**Figure 1:**
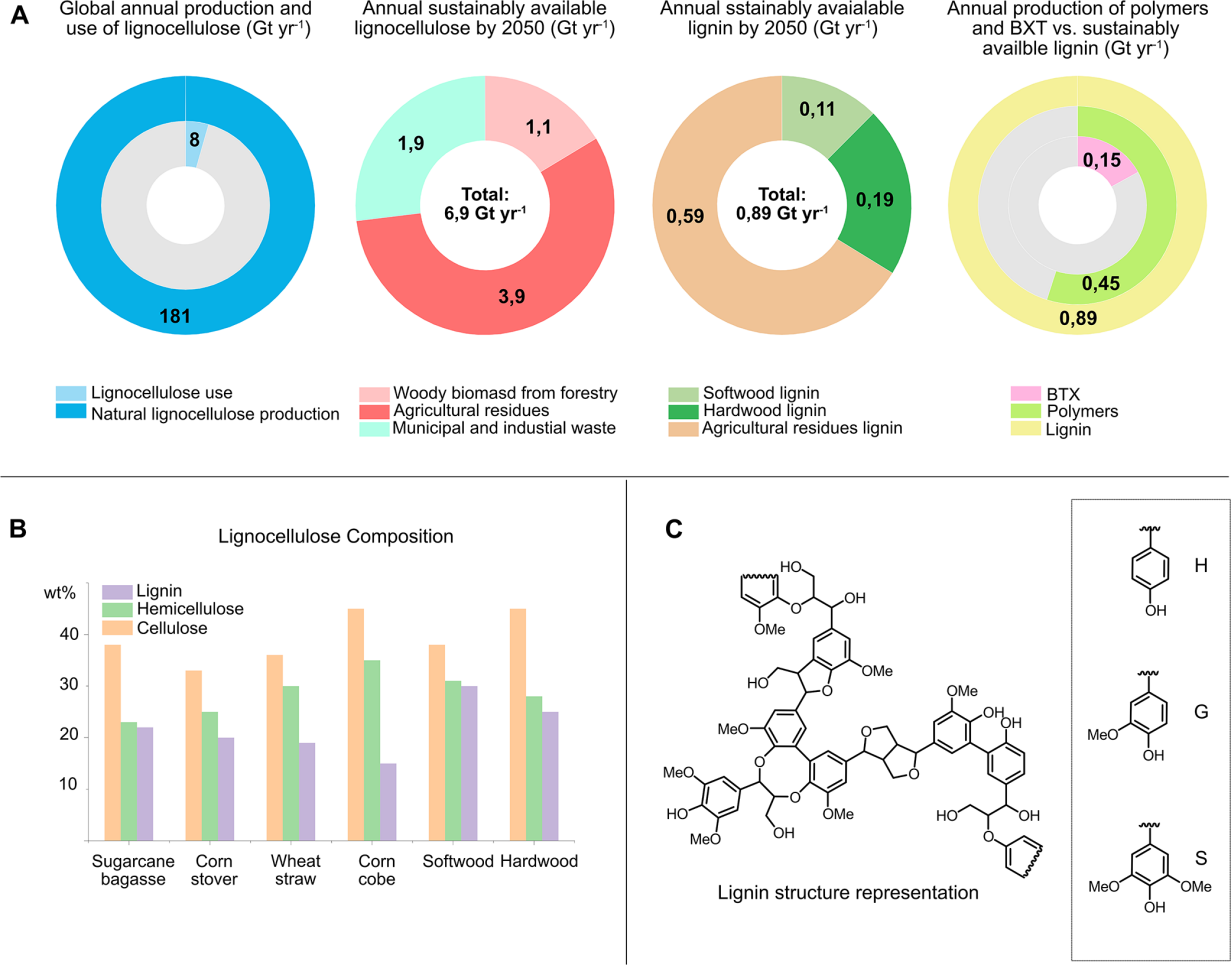
Global availability, composition, and structure of lignocellulosic biomass and lignin. (A) Availability of lignocellulose and lignin, including projected sustainable production in 2050, compared with current annual polymer production and BTX (benzene, toluene, xylene) production. (B) Composition of major lignocellulosic feedstocks. (C) Structural representation of lignin and its principal structural motifs.

### Lignocellulose Content and Composition

The composition of lignocellulosic biomass varies strongly with its biological origin, affecting both lignin content and structure. Forestry residues are predominantly wood-based and can be broadly classified into softwoods (gymnosperms) and hardwoods (angiosperms), which differ in vascular architecture and plant morphology: hardwoods contain vessel elements and form broad leaves (e.g., poplar, birch), whereas softwoods lack vessels, rely on tracheids for water transport, and typically bear needles (e.g., pine, spruce, fir). Globally, approximately 33% of industrially used wood originates from softwoods and 67% from hardwoods.^16^ Agricultural residues are mainly herbaceous biomass (e.g., corn stover, wheat straw, sugarcane bagasse), derived from nonwoody plants that typically die back at the end of growing season. As summarized in Figure 1B, softwoods exhibit the highest average lignin content (∼29.5 wt %), followed by hardwoods (∼25 wt %).^17^ The lignin content of agricultural residues varies by crop;^18^ here, a conservative average of 15 wt % (dry basis) is assumed.^19^ Based on the estimated sustainably available lignocellulosic biomass by 2050, the total lignin availability is approximately 0.89 Gt yr^–1^, comprising ∼0.11 Gt yr^–1^ from softwood residues, ∼0.19 Gt yr^–1^ from hardwood residues, and ∼0.59 Gt yr^–1^ from agricultural residues.

Another important aspect of lignin-to-monomers processes is the composition of the principal structural motifs in lignin. Lignin is a macromolecule which is biosynthesized via radical coupling of three aromatic monolignols—*p*-coumaryl, coniferyl, and sinapyl alcohols—giving rise to the corresponding *p*-hydroxyphenyl (H), guaiacyl (G), and syringyl (S) units differing in number of *o*-methoxy groups (Figure 1C). Softwood lignin is dominated by G units with negligible S content (typically S/G < 0.1), whereas hardwood lignin is enriched in S units (≈60–70% S; S/G ≈1.5–2.3). Herbaceous lignins exhibit the greatest compositional heterogeneity, containing appreciable amounts of H, G, and S units. For example, wheat straw lignin exhibits an approximate H:G:S ratio of 0.19:1:0.9.^20^

Because most lignin deconstruction strategies preserve the aromatic core (i.e., the number of methoxy substituents), the feedstock composition inherently governs the product distribution and attainable selectivity. Consequently, softwood lignin typically affords the highest monomer selectivity (G-unit derived products), whereas herbaceous lignocellulose yields the most complex and least selective product mixtures.

## From Lignin to Platform Molecules

The first step in lignin-to-monomers-to-polymers pathways is the conversion of lignin from a macromolecular biopolymer into streams of well-defined monoaromatic compounds referred here to as primary building blocks or platform molecules. Before discussing this transformation, we first define the nature of the lignin considered here. In the literature, lignin is often described as a side-stream of the pulp and paper industry; indeed, technical lignins constitute the largest industrially isolated lignin streams, including kraft lignin (∼78 Mt yr^–1^) and lignosulfonates (∼1.1 Mt yr^–1^).^21^ This Perspective, however, does not cover technical lignins and instead focuses on native lignin embedded in biomass (e.g., wood, grasses, corn cobs, sugarcane bagasse, and other agricultural residues). The rationale is the substantially more favorable structure of native lignin, which is enriched in cleavable ether linkages (carbon–oxygen bonds) compared to technical lignins that are dominated by recalcitrant carbon–carbon bonds. This structural distinction enables more efficient deconstruction and access to significantly higher yields of discrete lignin-derived aromatic building blocks, a prerequisite for viable lignin-to-monomers-to-polymers pathways. As such, native lignin available from forestry and agricultural residues is not only present in substantially larger quantities (0.89 Gt yr^–1^) but also offers markedly higher potential for conversion into defined monomers for polymer synthesis. Accordingly, the prevailing consensus in the field of lignin valorization considers native lignin the preferred substrate for the development of lignin-to platform molecules conversion.^22^

Two major conceptual approaches to lignin depolymerization have emerged. The most developed and widely applied is the lignin-first approach, which avoids prior isolation of lignin and instead promotes its selective fragmentation into well-defined low-molecular-weight compounds during biomass fractionation. In this strategy, lignocellulosic biomass in its native form (e.g., wood, grasses, straw) undergoes simultaneous fractionation into its primary components—cellulose, hemicellulose, and lignin—with lignin subsequently converted into platform molecules in situ.

An alternative strategy stabilizes lignin in its macromolecular form during fractionation, followed by isolation of a native-like lignin that can be selectively depolymerized into well-defined aromatic building blocks. This two-step approach decouples fractionation from depolymerization while preserving key structural features required for controlled bond cleavage. A major advance in this area was introduced by Luterbacher’s group through aldehyde-assisted fractionation (AAF) (Scheme 1).^23–25^ In this method, aldehydes form cyclic acetals with the α- and γ-hydroxyl groups of the phenylpropanoid units in lignin, thereby suppressing elimination of the α-hydroxyl group and preventing formation of highly reactive benzylic carbocations that drive undesired C–C bond formation during isolation. AAF enables lignin isolation under relatively mild, conventional pretreatment conditions while retaining high yields of both hemicellulose and cellulose. Importantly, the carbohydrate fractions remain free of transition-metal contamination, which can occur in lignin-first processes. The stabilized lignin can subsequently be depolymerized (e.g., by hydrogenolysis) to monophenolic products in yields comparable to the highest reported to date. Moreover, varying the stabilizing aldehyde allows tuning of lignin solubility and solvent choice, providing additional control over product selectivity.^26^

**Scheme 1:**
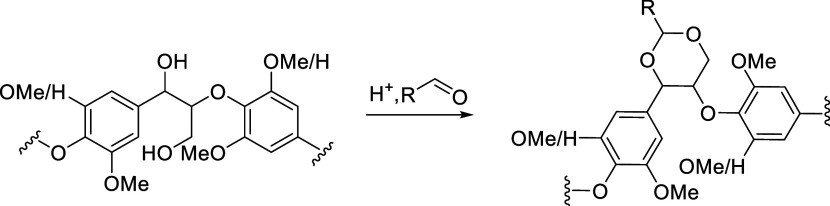
Stabilization of Lignin During Fractionation via Aldehyde-Assisted Fractionation (AAF); Acetal Formation at the α- and γ-Positions Preserves Native Structure of Lignin

While isolation of native-like lignin remains an active research area,^27,28^ this Perspective focuses primarily on lignin-first strategies, which are currently the most academically mature and widely studied. We do not aim to comprehensively cover the full spectrum of existing depolymerization methods (e.g., photo- and electrochemical approaches), which have been reviewed elsewhere.^29–36^ Instead, we focus on representative and comparatively developed strategies that collectively span the accessible range of lignin-derived products and illustrate the breadth of polymer-relevant building blocks currently available.

## Reductive Catalytic Fractionation (RCF)

Reductive Catalytic Fractionation (RCF) is one of the most well-studied methodologies for the production of platform chemicals from lignin.^22,33,35,42^ As the name suggests, RCF relies on the reductive cleavage of C–O bonds in lignin, resulting in the formation of phenolic monomers such as *p*-substituted guaiacols and syringols, often with high selectivity under optimized conditions (Scheme 2). RCF processes generally employ transition-metal catalysts (e.g., Ru, Pd, Cu), typically in combination with hydrogen gas (although hydrogen-free variants also exist), and polar solvent systems such as alcohol/water mixtures. These conditions enable simultaneous lignin extraction and cleavage in a single step, consistent with the lignin-first biorefinery concept. Importantly, the achievable monomer yields are intrinsically limited by the abundance of cleavable C–O linkages in the lignin structure; accordingly, hardwood lignin, which are richer in such linkages, affords the highest product yields, reaching up to ∼40 wt % under optimized conditions, whereas yields from softwood lignin are typically below ∼20 wt %. Within the scope of RCF, we focus on three representative families of processes that enable the production of reduced aromatic platforms bearing propyl-, 3-hydroxypropyl-, or propenyl-substituted side chains.

**Scheme 2:**
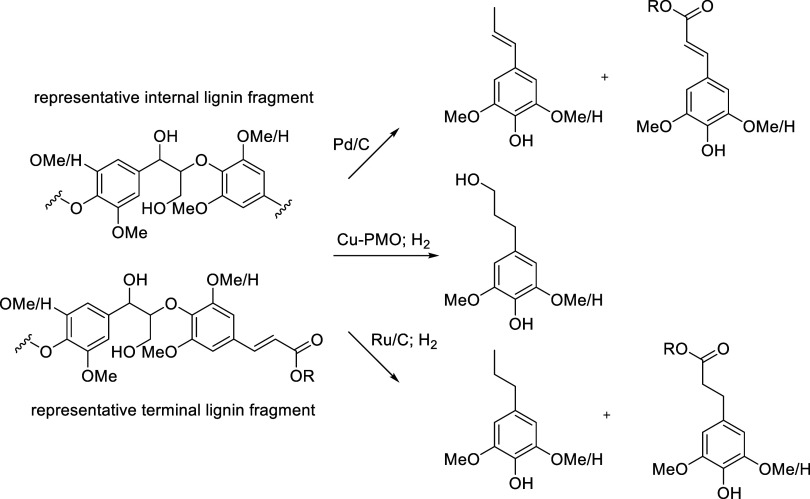
Products Obtained from Internal (β-*O*-4-linked) and Terminal Lignin Units via Ru/C–, Cu-PMO–, and Pd-catalyzed Hydrogen-Free Reductive Catalytic Fractionation (RCF)

In addition to lignin-derived products, RCF inherently generates streams of carbohydrates derived from the cellulose and hemicellulose fractions. The RCF process produces solid carbohydrate pulp and an aqueous phase containing sugars, polyols, and other products of carbohydrate decomposition. While RCF pulp generally exhibits lower fiber quality and strength metrics than kraft pulp optimized for high-performance paper applications, it remains amenable to downstream hydrolysis and conversion into value-added chemicals (bioethanol, levulinic acid, 5-hydroxymethylfurfural, etc.), offering added valorization pathways within a lignin-first biorefinery context.

### Ru/C-Catalyzed RCF

Ru-catalyzed RCF of wood is one of the most established methods for lignin valorization, originally developed primarily by Sels’ group.^43–45^ From wood, this process affords propylguaiacol and propylsyringol as the major products with high selectivity, alongside smaller amounts of ethyl- and propanol-substituted derivatives as byproducts. When applied to herbaceous lignocellulose, the product scope broadens to include dihydro-*p*-coumaric and dihydroferulic acid derivatives as major components. More recently, Beckham’s group conducted a comprehensive comparative study of Ru/C RCF applied to woody and herbaceous feedstocks (agricultural residues) under both batch and flow conditions.^37^ Using representative products yields from this study, the overall potential of this methodology can be estimated (Figure 2). Assuming that all sustainably available lignocellulosic biomass by 2050 were processed via Ru/C-catalyzed RCF, propylguaiacol production could reach approximately 48 Mt yr^–1^, while propylsyringol could reach ∼61 Mt yr^–1^, based reported monomer yields. In addition, substantial quantities of dihydro-*p*-coumaric (∼50 Mt yr^–1^) and dihydroferulic acids derivatives (∼39 Mt yr^–1^) could be generated. Thus, Ru-catalyzed RCF of forestry residues predominantly yields reduced, monofunctional products that exhibit enhanced stability but are not suited for direct use as polymer building blocks and would require secondary modification processes to be converted into multifunctional monomers; in contrast, in the case of agricultural residues, the process also yields bifunctional monomers such as dihydro-*p*-coumaric and dihydroferulic acids, which can potentially be used directly for polymer synthesis.

**Figure 2:**
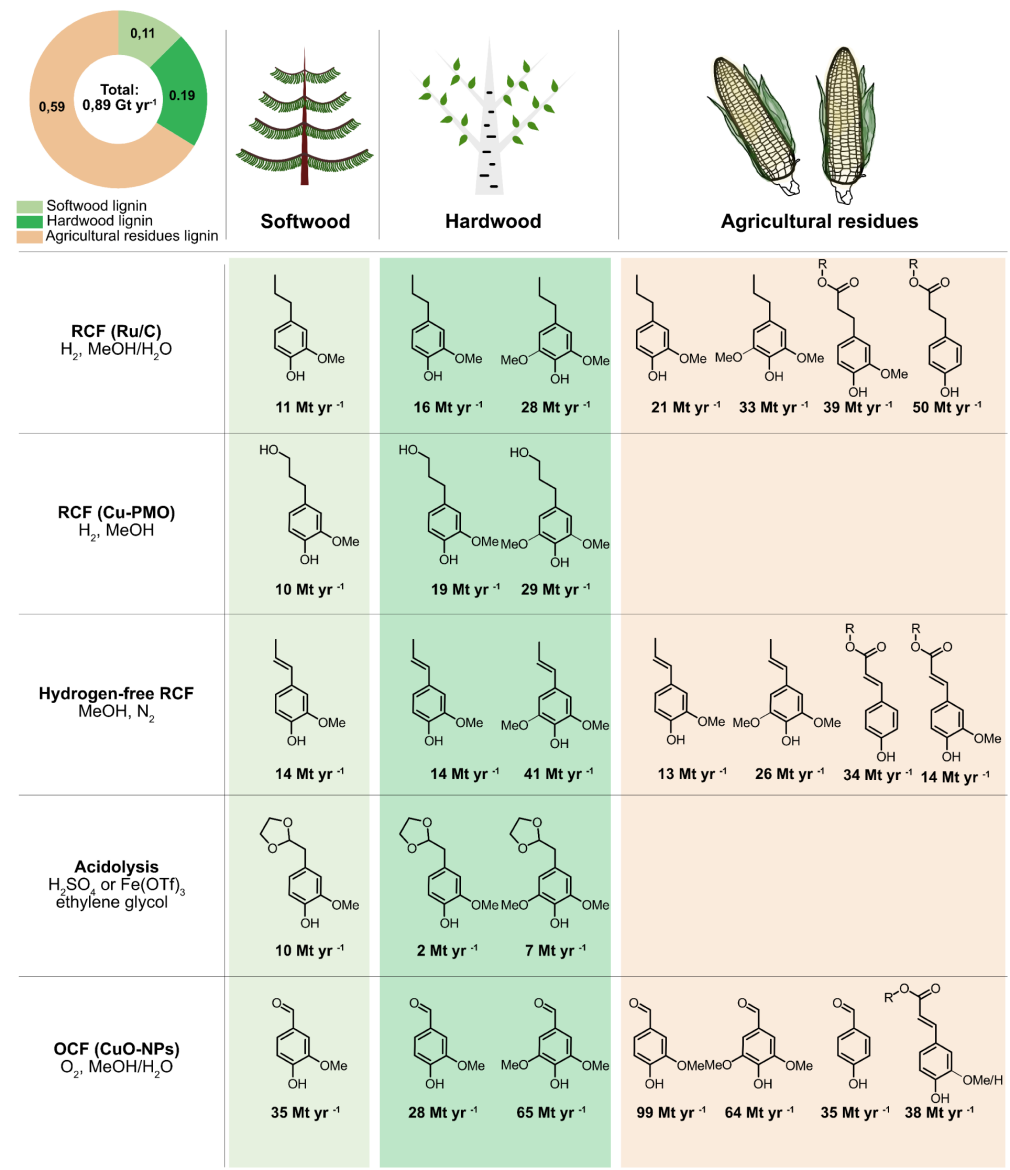
Major lignin-to-platform-molecule strategies and their estimated potential production volumes. Estimated production volumes for reductive catalytic fractionation (RCF; Ru/C–H_2_, hydrogen-free, and Cu-catalyzed variants), diol-stabilized acidolysis, and oxidative catalytic fractionation (OCF), assuming that all lignin of the corresponding type sustainably available by 2050 is processed via the respective pathway. Note: Calculations are based on representative literature reports: Ru/C RCF^37^ (Table S2 in ref. 37), Cu-PMO RCF^38^ (Supplementary Table S2 in ref. 38), hydrogen-free RCF^39^ (Table S10 in ref. 39), acidolysis softwood^40^ (Table 2 in ref.^40^), hardwood^27^ (Table 3 in ref. 27), and OCF^41^ (Table 2 in ref. 41), which report the yields of the corresponding products.

### Cu-Catalyzed RCF

This variant of RCF was largely developed by Barta’s group^38^ and enables the selective formation of products bearing a 3-hydroxypropyl side chain, such as 3-hydroxypropyl guaiacol and 3-hydroxypropyl syringol. The process typically employs porous copper oxide catalyst (Cu-PMO)^46,47^ in methanol in the presence of hydrogen gas. Although similar products can also be obtained with noble-metal catalysts such as Pd/C,^45^ the Cu-catalyzed system is particularly attractive due to the higher abundance and lower cost of copper-based catalysts.

Using data from representative study on Cu-PMO-catalyzed RCF,^38^ Figure 2 presents estimates for the annual production potential of these products from softwood and hardwood lignin, assuming that all sustainably available lignin from these feedstocks is utilized in the process. Under this assumption, the total annual production of 3-hydroxypropyl guaiacol and 3-hydroxypropyl syringol is estimated to reach approximately 29 Mt yr^–1^ for both (note that these estimates do not include contributions from agricultural residues due to the lack of corresponding studies). These values are comparable to those obtained for Ru-catalyzed RCF systems. However, in contrast to Ru-based RCF, the Cu-catalyzed variant delivers bifunctional molecules as primary products, rendering them more readily amenable to polymer synthesis^48^ or diversification into a broad range of value-added derivatives.^49,50^ Another distinct feature of this process is its approach to valorizing the carbohydrate fraction obtained after separation of the lignin-derived products. The residual carbohydrate pulp can be directly converted into mixtures of aliphatic alcohols under supercritical methanol conditions, a process that simultaneously enables efficient catalyst separation because no solid residues are formed. These alcohol mixtures can be further upgraded into cycloaliphatic hydrocarbons through consecutive dehydrogenation/aldol condensation and reduction steps.

### Hydrogen-Free RCF

Hydrogen-free RCF represents another important variant of lignin-first processing and predominantly yields propenyl-substituted phenolic products. Early development of this methodology was led by Samec’s group,^51^ which demonstrated a hydrogen-free Pd/C catalytic system capable of producing isoeugenol and propenyl syringol as major products under optimized conditions. A more recent example is provided by Liao’s group,^39^ which reported a low Ru loading catalytic system in which a pronounced shift toward propenyl-substituted products was observed when reactions were conducted under a nitrogen atmosphere rather than hydrogen. In this study, RCF was applied to softwood, hardwood, and agricultural residues (corn stover), providing a basis for estimating the potential production of propenyl-based platforms. Assuming that all sustainably available lignocellulosic feedstock of each type is utilized, the total annual production of isoeugenol is estimated at approximately 41 Mt yr^–1^, while propenyl syringol could reach 67 Mt yr^–1^. In addition, substantial quantities of *p*-coumaric- and ferulic acid-derived products are estimated at approximately 34 Mt yr^–1^ and 14 Mt yr^–1^, respectively. Overall, this process is beneficial because it eliminates the need for hydrogen gas and opens access to another family of multifunctional building blocks that combine olefinic functionality with a phenolic moiety. However, the inherently higher reactivity of the propenyl side chain compared to propyl or hydroxypropyl substituents may necessitate more careful optimization of the reaction conditions and handling of the product mixtures.

## Diol-Stabilized Acidolysis

Acidolysis is one of the oldest approaches to lignin depolymerization, but early conventional implementations suffered from low monomer yields due to rapid condensation of highly reactive phenylacetaldehyde products.^52–55^ To address this intrinsic limitation, Barta and coworkers introduced an in situ protection strategy based on ethylene glycol acetalization, which suppresses condensation and stabilizes the products (Scheme 3).^56–58^ Implemented directly on softwood in a lignin-first configuration (H_2_SO_4_, ethylene glycol in dimethyl carbonate), this approach enabled highly selective formation of the ethylene glycol acetal of homovanillin, with an estimated production potential of ∼10 Mt yr^–1^ (Figure 2).^40^

**Scheme 3:**
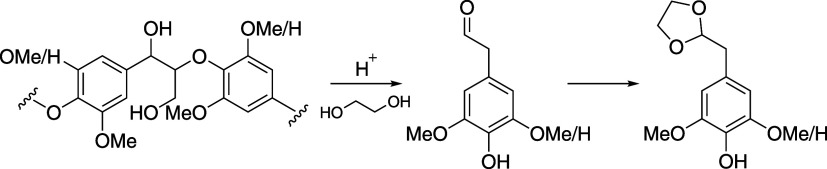
Products Obtained from Internal (β-*O*-4-linked) Lignin Units via Diol-Stabilized Acidolysis

In contrast, this process for hardwood has so far only been demonstrated via a two-step strategy, in which ethylene-glycol-protected native-like lignin is first isolated using deep eutectic solvents, followed by acidolysis with acetal stabilization.^27^ Although this approach enables lignin stabilization and allows its isolation in a more native-like form, lignin recovery remains nonquantitative (∼53 wt %), which limits overall monomer yields (combined acetals of homovanillin and homosyringaldehyde estimated at ∼9 Mt yr^–1^). While less productive than reductive strategies, this methodology uniquely provides functional Ar–C_2_ building blocks with high selectivity that are inaccessible by other lignin depolymerization routes and does not require transition-metal catalysts. Overall, this approach offers unique lignin-derived platforms but remains less mature than RCF technologies, particularly for hardwood feedstocks.

## Oxidative Catalytic Fractionation (OCF)

Oxidative depolymerization of lignin is another approach with long history and industrial relevance, most notably in the production of vanillin via alkaline oxidation of technical lignins such as kraft lignin^59,60^ and lignosulfonates.^61,62^ In contrast, oxidative depolymerization of native lignin has only recently been revisited in a lignin-first context, with examples including copper-based,^63,64^ uncatalyzed,^64^ and cobalt-catalyzed systems.^65,66^ This renewed interest has led to the development of oxidative catalytic fractionation (OCF) as a distinct lignin-first strategy (Scheme 4).

**Scheme 4:**
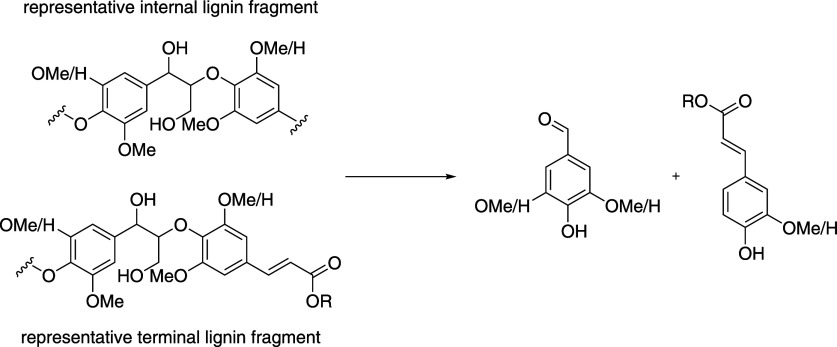
Products Obtained from Internal (β-*O*-4-linked) Lignin and Terminal Lignin Units via Oxidative Catalytic Fractionation (OCF)

OCF offers several advantages over reductive and acidolysis-based approaches, most notably enabling cleavage of both C–O and C–C bonds, thereby expanding the accessible product space and increasing the theoretical product yield. Moreover, OCF can be conducted under aerobic conditions using molecular oxygen or air and typically proceeds under relatively mild conditions. Despite these advantages, product overoxidation and secondary reactions have historically limited achievable yields. A recent comprehensive study comparing uncatalyzed and metal-catalyzed systems showed that Cu-based catalysts are particularly effective for native-like lignins.^67^

Building on these insights, Sels’ group recently developed a CuO nanoparticles (CuO-NPs)-catalyzed OCF system that enables the formation of aromatic aldehydes in high yields (up to 50 wt %) and with good selectivity.^41^ Using this approach, OCF was applied to softwood, hardwood, and agricultural residues (bagasse), resulting in an estimated total production potential of vanillin (∼162 Mt yr^–1^), syringaldehyde (∼129 Mt yr^–1^), and *p*-hydroxybenzaldehyde (∼35 Mt yr^–1^), along with substantial quantities of *p*-coumaric- and ferulic acid derivatives (∼38 Mt yr^–1^ combined). These numbers underscore the strong potential of OCF, particularly when combined with the fact that the CuO-based system preserves up to ∼80% of the cellulose in high purity—a long-standing challenge due to competing oxidative degradation pathways. While this methodology is highly promising, it remains comparatively less explored and will require further study to assess its robustness, scalability, and broader applicability.

Lignin-first strategies currently offer the most selective and controlled routes to lignin-derived building blocks. The dominant products are *p*-substituted guaiacols, syringols, and hydroxyphenols, which differ in the structure of the side chain (most commonly C_1_ or C_3_). Notably, unsubstituted phenolic products (phenol, guaiacol, syringol), although often cited as lignin-derived, are not directly accessible under lignin-first conditions; their formation requires harsher downstream transformations (e.g., dealkylation and demethoxylation)^68,69^ or alternative, less selective pathways such as pyrolysis rather than controlled lignin deconstruction.

Feedstock choice strongly governs composition of products mixtures. Softwood enables selective access to G-derived monomers, whereas hardwood yields mixtures of G- and S-derived products, necessitating downstream separation, direct use as mixtures, or funneling strategies to converge mixtures into a single product stream. Importantly, ferulic- and *p*-coumaric-acid derivatives (or their reduced counterparts) are obtained in meaningful quantities only from agricultural residues (e.g., corn stover, bagasse) and are not available at scale from forestry biomass.

Among lignin-first technologies, OCF currently delivers the highest monomer yields and uniquely enables partial C–C bond cleavage, but remains less mature and less broadly validated. In contrast, RCF is the most developed and scalable platform to date; however, it predominantly furnishes reduced, monofunctional products, albeit ones with high stability.

## From Lignin-Derived Platform Molecules to Monomers for Polymer Synthesis

In this section, we examine approaches for converting relevant lignin-derived building blocks into polymerizable monomers and subsequently into polymeric materials. One of the most straightforward and widely explored strategies for integrating lignin-derived platforms into existing industrial polymer value chains is to target high-volume aromatic polymers and develop their structural lignin-based congeners (drop-in substitutes). In this context, bisphenol A (BPA)-based polymers, polyethylene terephthalate (PET), and polystyrene (PS) represent the most prominent targets, together covering the largest share of the total aromatic polymer volume and accounting for ∼24% of global polymer production. In the following discussion we highlight selected examples illustrating progress toward these polymer targets.

### Lignin-Derived BPA Analogs

BPA-based materials are a large class of aromatic polymers, with global BPA production on the order of ∼6 Mt yr^–1^, primarily used in polycarbonates (∼66%) and epoxy resins (∼30%).^70,71^ The motivation to redesign BPA-derived polymers extends beyond the transition away from fossil resources: BPA is associated with well-documented adverse health effects,^72–74^ including metabolic disorders, reproductive toxicity and especially concerning endocrine disruption.^75^ As a result, toxicological performance is a central design criterion for next-generation BPA substitutes. Notably, several early replacements (e.g., bisphenol S, F, and AF) have shown comparable or even worse toxicological profiles than BPA,^76,77^ highlighting the need for fundamentally redesigned and carefully evaluated BPA alternatives rather than simple structural analogs, with lignin-derived phenolics providing a promising starting scaffold. One of the key reasons for the endocrine activity of BPA and many of its analogs is their affinity for estrogen receptors α and β (ERα/ERβ). Recent structure–toxicity relationship studies of BPA analogs have identified several structural features that reduce estrogen receptor binding potency and efficacy: (i) the presence of methoxy groups *ortho* to the phenolic hydroxyl groups,^71,78,79^ (ii) deviation from the classical *para, para*′-arrangement of phenolic groups relative to the bridging unit,^80,81^ and (iii) incorporation of polar functionalities within the bridging group (Figure 3A). In this regard, lignin-derived building blocks are particularly promising, as they inherently possess *ortho*-methoxy substituents and typically feature blocked *para* positions relative to the phenolic hydroxyl groups, which disfavors formation of the *para-,para-*′-phenolic motif associated with high estrogenic activity. Moreover, both the bridging group type (i.e., number of carbon atoms) and the substitution pattern can be widely varied, providing access to a broad range of novel bisphenol architectures. This versatility has enabled the development of a wide range of structurally diverse lignin-derived bisphenol candidates.

**Figure 3:**
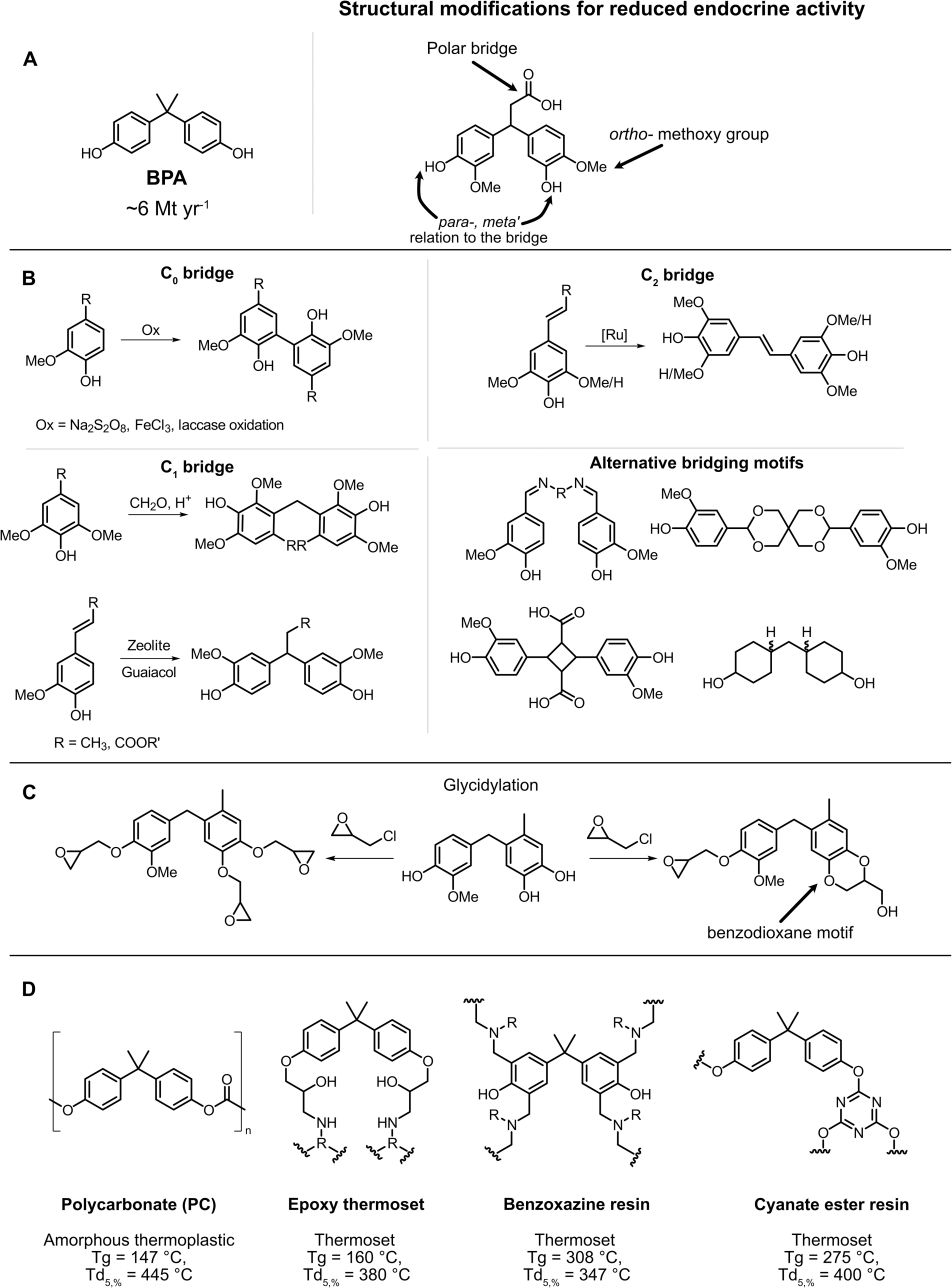
Lignin-derived substitutes for BPA-based materials. (A) Structure of BPA and representative structural modifications aimed at reducing its endocrine-disrupting activity. (B) Design space of lignin-derived BPA analogs. (C) Bis-catechols as tunable scaffolds for epoxy resins. D. Most common commercial BPA-based polymers and their thermal properties.

Figure 3B outlines this structural diversity, encompassing bisphenols without a bridging atom (C_0_) obtained via radical oxidative coupling of guaiacyl-derived platforms;^82–84^ C_1_-bridged architectures accessible via Friedel–Crafts-type hydroxyalkylation/alkylation using either external C_1_ sources (e.g., formaldehyde,^85–89^ levulinic acid,^90^ furfural^91,92^ ) or the benzylic carbon of lignin-derived substrates themselves (e.g., isoeugenol,^93,94^ ferulic/*p*-coumaric acid derivatives,^93^ vanillyl alcohol^78,95,96^ ); C_2_-bridged structures accessed via olefin metathesis of alkenyl-substituted lignin platforms;^97–100^ and a diverse set of structures with longer aliphatic, cyclic, and spirocyclic bridging motifs^101–103^ as well as cycloaliphatic bisphenols.^104^ This breadth underscores the suitability of the lignin-enabled design space for BPA congeners, as the combined demands of matching existing material properties and addressing toxicological concerns require flexible synthetic routes that provide rapid access to diverse libraries for materials properties optimization and toxicological screening. More importantly, such structural redesign allows polymer properties to be tailored to the requirements of specific applications, in contrast to BPA, which represents a single, broadly applied monomer platform. In redesigned systems, functionality required for a given application can be incorporated directly during monomer synthesis. For example, vanillin-based bisphenols can incorporate labile spiroacetal^101–103^ motifs that introduce controlled degradability or recyclability to facilitate end-of-life material recovery, while phosphonate^105^ or triazole groups^106^ impart intrinsic flame-retardant properties.

### Polymers Based on Lignin-Derived BPA Analogs

To evaluate the potential of lignin-derived building blocks as substitutes for commercial BPA-based polymers, it is instructive to benchmark their theoretical production capacities against the current global BPA market (≈6 Mt yr^–1^) and assess their ability to match the material properties required for established applications. A comprehensive techno-economic analysis would provide further insight into this evaluation but lies beyond the scope of the present perspective.

The estimates suggest that phenols obtainable from only a fraction of forestry residues would be sufficient to meet current market demand. Assuming an average bisphenol yield of 75% (consistent with literature reports), Ru/C-catalyzed RCF applied to 15% of sustainably available forestry residues (softwood and hardwood combined)—or hydrogen-free RCF applied to 12% of these residues—would generate bisphenols at a scale comparable to the current global BPA market. For OCF, the required fraction is even lower (∼6%). This makes it reasonable to consider a dedicated single-product strategy in which OCF is applied exclusively to softwood to produce vanillin with high selectivity, requiring approximately 23% of sustainably available softwood resources.

In terms of performance, most studies evaluating bioderived substitutes for BPA-based materials benchmark them against their commercial counterparts. Typical metrics include glass transition temperature (Tg), thermal stability (e.g., Td_5%_ and Td_50%_), and, where relevant to the target application, mechanical performance and water uptake. A common trend is somewhat lower thermal stability, likely associated with the higher degree of oxygenation in lignin-derived monomers. Tg values are also often reduced, partly due to the presence of methoxy groups^78,107^ and side-chain substituents, which increase free volume and hinder efficient chain packing.

In addition, the resulting polymers frequently deviate from the characteristic white appearance of conventional BPA-based products and instead exhibit a yellowish coloration. Whether such deviations are critical depends not only on the polymer class but also on the specific application sector—an aspect that is often insufficiently addressed in literature. Each polymer class must therefore be evaluated individually.

### Polycarbonates (PC)

Polycarbonates (PCs) are typically used in applications where transparency—arising from their amorphous structure—high impact strength, and thermal stability are critical. The latter is particularly important in defining a sufficiently wide processing window while avoiding thermal degradation or discoloration. PCs are therefore widely employed in protective and optical applications, including safety shields, visors, goggles, optical lenses, and safety glazing, where high transparency and impact resistance are essential. They are also commonly used in biomedical devices (e.g., syringes and medical housings), where resistance to thermal sterilization is required.

PCs prepared from lignin-based bisphenols generally exhibit the desired amorphous structure due to the presence of aliphatic substituents in the aromatic ring or in the bridging unit, which disrupt efficient chain packing.^87^ An exception is observed for PCs derived from bisphenols containing unsubstituted methylene bridges and lacking longer aliphatic substituents, which tend to crystallize.^89^ Importantly, although many lignin-derived PCs are transparent, they often exhibit a yellowish coloration. This may limit their suitability for certain applications (e.g., protective goggles) while remaining acceptable for others (e.g., tubing). Such application-specific aesthetic and performance considerations are rarely discussed in the literature, making it difficult to determine whether the observed coloration arises from suboptimal synthetic procedures or from more fundamental characteristics of lignin-derived bisphenols, such as increased susceptibility to thermal degradation.

In terms of thermal performance, lignin-derived PCs generally exhibit lower Tg values (100–115 °C) compared to BPA–PC (∼147 °C, Figure 3D),^71^ although cycloaliphatic variants with tunable Tg values up to 174 °C have been reported.^104^ Thermal stability is more significantly affected, with Td_5%_ values often 80–100 °C lower than BPA–PC,^71^ which may complicate processing at typical extrusion or injection-molding temperatures (≈280–300 °C). Ultimately, the relevance of these deviations can only be assessed within the framework of the intended application and processing window, as requirements for thermal stability, transparency, mechanical robustness, and durability vary substantially across sectors.

### Epoxy Resins

BPA-based epoxy resins are widely used in adhesives, coatings, and especially composites. Compared to PCs, epoxy systems are far more flexible: their properties can be tuned through the choice of curing agent, curing conditions, and cross-link density. In composite materials, the design tunability is even greater, as the reinforcing phase (e.g., fibers or fillers) can compensate for shortcomings in the epoxy matrix, mitigating limitations in thermal or mechanical performance when needed. This wide and tunable performance window makes lignin-derived epoxy resins particularly attractive BPA alternatives. Unlike PCs, which impose stringent requirements on transparency and thermal stability, epoxy and composite systems can tolerate greater structural variation in the monomer while still meeting application-specific performance targets—making them a more forgiving and impactful entry point for biobased bisphenols.

Biobased bisphenol analogs also enable the introduction of novel functionalities into epoxy thermosets. A representative example that leverages the inherent functionality of lignin-derived bisphenols comes from a series of studies by the Abu-Omar group.^88,96^ In this work, catechol-based bisphenols were obtained via demethylation of their guaiacol counterparts and subsequently employed to design high-performance epoxy materials. The introduction of additional phenolic hydroxyl groups as reactive handles increased the number of epoxy groups installed via glycidylation with epichlorohydrin, which in turn enhanced the cross-link density of the cured networks. This was reflected in significantly improved thermal stability, with Td_5%_ values reaching up to 297 °C (Figure 3C). Furthermore, the authors demonstrated that the network structure of these systems can be tuned to yield either rigid, strong materials or more ductile, reconfigurable networks. Depending on the reaction conditions, treatment of the catechol-containing substrates with epichlorohydrin can yield either diglycidyl ethers or induce intramolecular cyclization to form benzodioxane structures bearing dangling hydroxyl groups (Figure 3C). The ratio between epoxide functionalities and benzodioxane units governs both the cross-link density and the concentration of pendant hydroxyl groups in the cured material. The presence of these hydroxyl groups enables exchange reactions within the network, imparting vitrimer-like behavior to the epoxy thermosets. As a result, the network topology becomes reconfigurable under appropriate thermal or catalytic conditions, allowing the materials to be reshaped or reprocessed while maintaining the mechanical robustness characteristic of conventional thermosets.

Important to note that while catechol-containing lignin-derived building blocks have attracted considerable research interest, demethylation strategies require further development to render catechols more accessible from lignin through milder and less toxic transformations. Current methods typically rely on toxic reagents such as BBr_3_, HBr, or related systems, which limit scalability and sustainability.^108^

### Cyanate Ester Resins and Benzoxazine Resins

Other BPA-derived polymer classes—such as cyanate ester resins, benzoxazine resins, and polyarylates—are produced at significantly lower volumes than epoxy resins and PCs and are typically classified as engineering polymers for high-performance applications (e.g., aerospace components, advanced composites, electronic encapsulation, and flame-retardant systems). In these sectors, compliance with stringent performance requirements constitutes the primary criterion for material selection. Consequently, the development of lignin-derived substitutes for BPA-based systems must prioritize matching—or ideally surpassing—the performance of established benchmark materials to provide a compelling motivation for substitution.

A key question is whether intrinsic structural features of lignin-derived building blocks can be strategically leveraged to enhance performance or broaden the scope of applications. Benzoxazine resins provide a representative example. One of their key performance metrics is char formation, reflecting their widespread use in fire-critical environments such as aerospace structures, electronic encapsulation, and flame-retardant coatings. Lignin-derived benzoxazine resins frequently exhibit higher char yields compared to their BPA-based counterparts.^91,109,110^ This enhancement is commonly attributed to methoxy substituents on the aromatic ring. Upon thermal activation, these groups promote the formation of reactive *o*-quinone intermediates, which can undergo secondary reactions—such as Diels–Alder cycloadditions—leading to the formation of highly condensed aromatic structures and, ultimately, increased char formation.^111^ In this context, methoxy groups are clearly advantageous.

In contrast, methoxy groups are detrimental in cyanate ester resins. For these materials, moisture resistance is a critical performance parameter because cured networks contain cyanurate linkages that are susceptible to hydrolytic degradation.^112–114^ Methoxy substituents have been shown to significantly increase water uptake^115^—by up to ∼40% in some systems^116^—thereby facilitating hydrolysis and compromising long-term stability. Incorporation of propyl side chains can partially mitigate this effect, as demonstrated for cyanate ester resins derived from propyl guaiacol derivatives, where hydrophobic propyl groups reduce moisture diffusion and improves hydrolytic stability.^86^ Given the strong moisture sensitivity of cyanate ester resins, monomer design should prioritize minimizing of water uptake in the cured network. Rather than offsetting the negative impact of methoxy groups with propyl substituents, a more practical strategy may be to eliminate methoxy groups altogether and directly employ *p*-propyl phenolic building blocks. Such structures could be obtained from lignin-derived propyl guaiacol or syringol intermediates via demethoxylation.^68,69^ Although this step is energy-intensive, it may be justified in this case given the lower production volumes and stringent performance requirements of cyanate ester resins.

### Pathways toward PET Analogs

Polyethylene terephthalate (PET) represents another major commercial polymer targeted for lignin-derived substitution, with global production of approximately 82 Mt yr^–1^.^117^ PET is a semicrystalline polymer, but its crystallization rates are relatively low, allowing access to both amorphous and semicrystalline materials depending on processing conditions. Amorphous PET is primarily used in beverage bottles and packaging, whereas semicrystalline PET is predominantly employed in fibers and textiles.

In contrast to bisphenols-, terephthalic acid bears less direct structural resemblance to typical lignin-derived platforms, resulting in a broader diversity of synthetic strategies toward PET-relevant monomers. These monomers can be classified according to step-growth polymerization notation (e.g., A-A and A-B) and by functional group type (aromatic (Ar–COOH), aliphatic (Alkyl-COOH), and α,β unsaturated (Alkenyl-COOH)­carboxylic acids; and phenols (Ar–OH)/aliphatic alcohols (Alkyl–OH)). Representative examples of each category reported in the literature are summarized in Figure 4.

**Figure 4:**
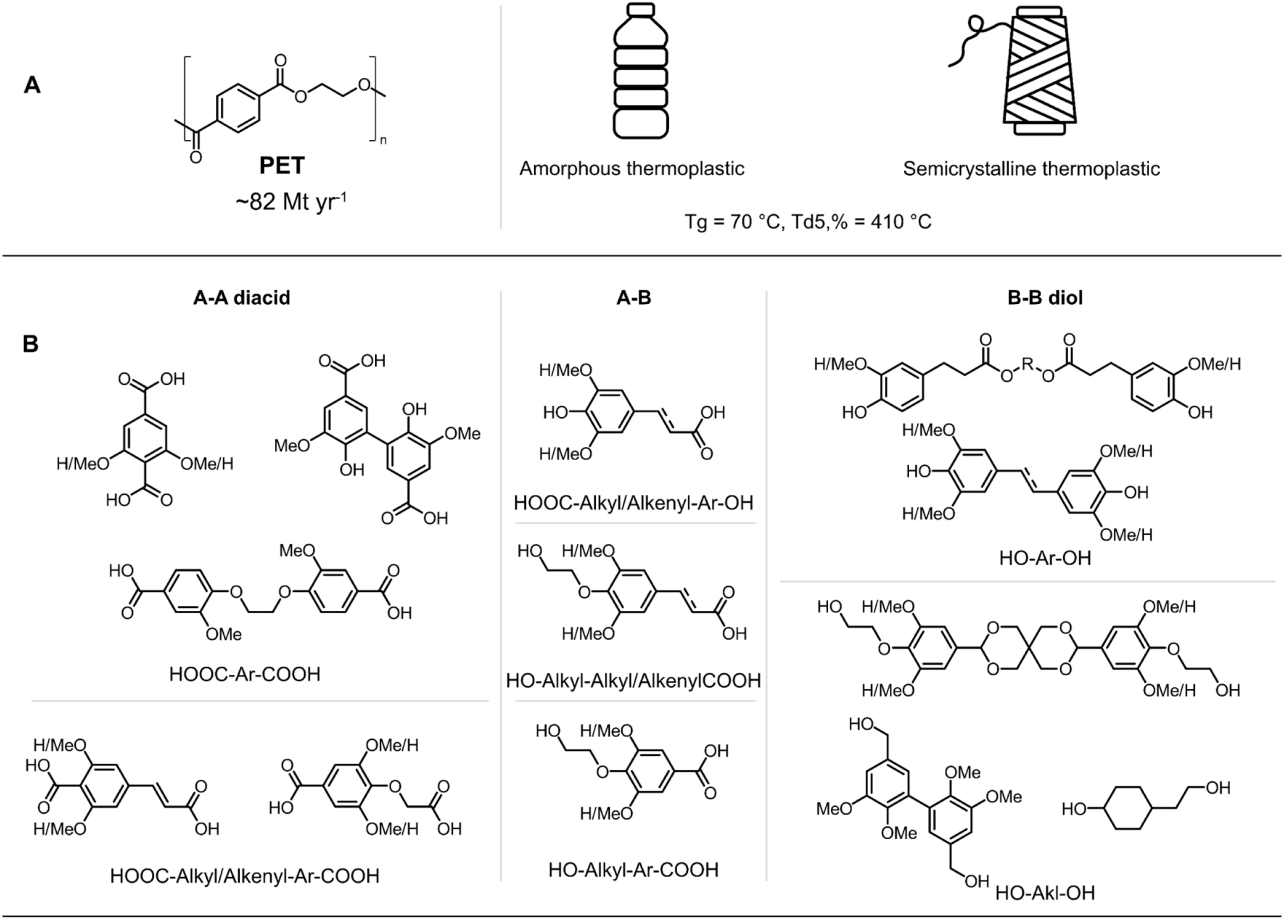
Lignin-derived substitution pathways toward PET. (A) Chemical structure of polyethylene terephthalate (PET) and its major commercial applications. (B) Chemical space of lignin-derived aromatic monomers accessible for the development of PET analogues and related copolymers.

### HOOC–Ar–COOH (A-A Type)

The most straightforward strategy to access lignin-based monomers for PET production is direct synthesis of terephthdalic acid (TPA) from lignin-derived platforms.^118,119^ This approach involves the conversion of phenolic functionalities present in lignin platforms into aryl carboxyl groups. A number of established transition-metal-catalyzed methodologies enable this transformation,^120^ including reductive electrochemical Ni-catalyzed carboxylation,^121^ Pd/Ir-mediated photoredox carboxylation,^122,123^ and Pd-catalyzed carbonylation via CO insertion into the aryl–O bond.^119^ Subsequent demethoxylation is required to obtain terephthalic acid. Additionally, depending on the initial lignin valorization process, further oxidation of alkyl side chains (e.g., propyl groups) may be necessary, particularly when monomers are derived from RCF pathways.^118^ Reported average yields of terephthalic acid, calculated based on the initial lignin-derived platforms (either realistic mixtures or model compounds), are approximately 45%.

Within this direct substitution strategy, the performance of the resulting PET is expected to be comparable to that of commercially available material, as its properties are only governed by the purity of the synthesized TPA. The feasibility of such a substitution therefore depends primarily on achieving sufficient production volumes and on techno-economic viability (i.e., production cost relative to market price). Assuming quantitative conversion of TPA and ethylene glycol into PET via polycondensation, supplying a global demand of 82 Mt yr^–1^ of PET would require approximately 65 Mt yr^–1^ of TPA. Unlike BPA-based materials, this volume cannot be obtained exclusively from forestry residues processed via either RCF or OCF; agricultural residues would also need to be incorporated as feedstock. In the RCF scenario, meeting the required TPA demand would consume approximately 73% of all sustainably available lignin projected for 2050 (based on Figure 2), assuming an average 45% yield for conversion of monomer mixtures into TPA. In the OCF case, the corresponding share is approximately 20%. These fractions are already substantial and may increase further when more complex realistic monomer mixtures are considered. Importantly, assuming that such a large proportion of all global biomass resources would be processed through a single technological pathway is unrealistic. Both RCF and OCF are primarily designed for lignin valorization and may not be optimal for the efficient recovery and utilization of other biomass components, such as cellulose and hemicellulose. Furthermore, the reliance on multiple transition-metal-catalyzed steps adds additional technical and economic burden, reinforcing the conclusion that complete substitution of conventional fossil-based PET production via this route remains highly challenging.

Another important general aspect to consider when developing lignin-based PET analogs is that alternative biobased platforms may, in some cases, offer higher overall potential. For example, polyesters based on furan dicarboxylic acid (FDCA),^124^ which can be produced from the carbohydrate fraction of lignocellulosic biomass (typically ∼70–85% of the total biomass, compared to ∼15–30% for lignin), have demonstrated very promising material properties. In particular, FDCA-based polyesters exhibit excellent gas barrier performance, making them especially attractive for applications such as beverage bottles. Although these materials lie outside the scope of this Perspective, it is important to keep such competing biobased polymer platforms in mind when assessing the broader sustainability and application potential of lignin-derived PET analogs.

In light of these constraints, it is more compelling to move beyond a strict direct-substitution strategy and instead explore structures that more closely resemble native lignin-derived platforms. In a comprehensive study, Gregg Beckham’s group investigated the polymerization behavior of guaiacyl- (monomethoxy terephthalic acid) and syringyl- (dimethoxy terephthalic acid) derived monomers.^125^ Both were found to polymerize less efficiently than unsubstituted TPA, with the effect particularly pronounced for syringyl-type monomers. This behavior is likely attributable to steric hindrance imposed by the ortho-methoxy substituents, which restrict access to the carboxyl functionalities and thereby limit polymer growth. However, these lignin-derived monomers proved effective when used as drop-in comonomers at low incorporation levels (up to ∼10 mol %). Within this compositional window, PET-like glass transition temperatures (∼70 °C) and high thermal stability (Td5% ≈400 °C) were maintained. In this regime, mono- and dimethoxy TPA moieties function analogously to isophthalic acid in conventional PET formulations: they reduce crystallinity and lower the melting temperature, thereby improving processability without significantly compromising bulk mechanical or thermal properties. Beyond processing advantages, methoxy substitution may also influence end-of-life performance. Incorporation of these monomers modifies the hydrolytic depolymerization behavior of PET. The ortho-methoxy groups exert an inductive electron-withdrawing effect on the adjacent carbonyl carbon, increasing its electrophilicity and thereby facilitating nucleophilic attack during hydrolysis. This electronic activation of the ester linkage could potentially enhance the efficiency of chemical recycling routes for PET.^125^ Compared to complete TPA substitution, this partial comonomer incorporation strategy represents a more realistic and technically feasible approach for integrating lignin-derived aromatic platforms into PET-based materials.

Overall, the development of strategies that convert lignin-derived phenolic monomers into aromatic dicarboxylic acids is scientifically compelling, as it enables access to new aromatic monomers and significantly expands the chemical space of lignin-based building blocks. However, despite this conceptual attractiveness, translating such approaches into large-scale substitutes for conventional PET production remains highly challenging of volume requirements and economic feasibility.

### HO–Ar-Alkyl-COOH (A-B Type)

Another widely explored class of lignin-based polyesters that mimic PET, pioneered by Miller’s group, is based on dihydroferulic acid.^126^ This compound is the hydrogenated form of ferulic acid—one of the platform molecules derived from herbaceous lignocellulose—and is also readily accessible from vanillin via a Perkin or Knoevenagel–Doebner condensation. Rather than directly replicating petroleum-based PET production, this approach introduces structurally modified monomers that enable the formation of new polyesters with comparable, and potentially advantageous, properties. In contrast to conventional PET synthesis via A-A + B-B step-growth polycondensation—which requires precise stoichiometric balance between diacid and diol monomers to achieve high molecular weight—dihydroferulic acid functions as an A-B-type monomer containing both a carboxylic acid and a phenolic hydroxyl group within the same molecule. This intrinsic functional group balance simplifies polymerization and reduces sensitivity to monomer feed ratios, facilitating high conversion and molecular-weight development. The resulting polymers are structurally distinct from PET. They contain ester linkages that are more susceptible to hydrolytic cleavage due to the superior leaving-group ability of phenols compared to aliphatic alcohols, which is relevant for end-of-life chemical recycling. This aspect, however, requires careful examination to ensure that the increased susceptibility to hydrolysis does not compromise performance during the use phase. The polymers derived from dihydroferulic acid exhibit favorable thermal properties, with a Tg of approximately 73 °C and a melting temperature (Tm) of about 234 °C.^126^ For applications where amorphous PET is desirable (e.g., transparent packaging), performance is primarily governed by Tg. In this regard, the Tg of these lignin-derived polyesters (∼73 °C) is comparable to that of PET (∼70 °C), indicating similar upper service temperatures without significant compromise in performance. For applications requiring semicrystalline materials, such as fibers, Tm becomes more relevant. Although the Tm (∼234 °C) is lower than that of PET (265 °C), it remains sufficiently high for most end-use conditions, which rarely exceed 200 °C. At the same time, the reduced Tm can be advantageous, as it facilitates melt processing and fiber spinning without sacrificing thermal stability during use.

### HO-Alkyl–Alkyl/Alkenyl-COOH (A-B Type)

In the previously discussed strategy, a rigid aromatic ring was combined with the flexible aliphatic side chain of ferulic acid to approximate the structural balance found in PET, where aromatic terephthalate units are linked by flexible ethylene glycol segments. Building on this concept, Miller’s group further expanded the design space by systematically exploring a broader family of lignin-derived monomers, including hydroxyalkylated ferulic, dihydroferulic, *p*-coumaric, and dihydro-*p*-coumaric acids.^127^

Through controlled variation of monomers mixture composition, the resulting polyesters exhibit Tg spanning a wide range, from 32 to 134 °C. This range encompasses materials comparable to PET as well as higher-Tg polymers such as polystyrene (PS; Tg ≈ 100 °C), highlighting potential applications beyond direct PET substitution. Clear structure–property relationships were identified in this systematic study. Methoxy substituents lead to a modest decrease in Tg, whereas unsaturated side chains, as present in ferulic and *p*-coumaric acids, significantly increase Tg due to enhanced chain rigidity. In contrast, incorporation of additional flexible aliphatic segments through hydroxyalkylation markedly lowers Tg by increasing chain mobility. These results highlight that, rather than viewing the compositional diversity of lignin-derived monomers as a limitation, it can be leveraged as a design parameter, enabling controlled tailoring of polymer properties through compositional tuning.

### HO-Alkyl-Ar–COOH (A-B Type)

As a final variation of this approach, Miller’s group explored bifunctional monomers containing aromatic carboxyl and aliphatic hydroxyl groups, specifically hydroxyethylated *p*-hydroxybenzoic, vanillic, and syringic acids.^128^ These monomers afforded polyesters with number-average molecular weights of 14,000–23,000 g·mol^–1^ and Tg between 66 and 80 °C, with Tg decreasing as the number of methoxy substituents increased. Important to note, this strategy enabled efficient incorporation of syringic units. In contrast to earlier approaches using dimethoxy terephthalic acid,^125^ which yielded low molecular weight polymers due to steric hindrance of the carboxyl group, hydroxyethylated syringic acid polymerized more effectively. Relocating the reactive hydroxyl group onto an aliphatic spacer reduces steric congestion and facilitates polymer growth.

The direct substitution strategy discussed earlier—namely, the production of TPA from lignin-derived aromatics—seems problematic in terms of matching monomer production volumes to meet the projected demand and is not feasible if only forestry residues are considered. In contrast, the A-B-type monomer approach described above offers significantly improved material efficiency. Considering OCF applied to forestry residues, the resulting streams of vanillin and syringaldehyde can be converted either into the corresponding aromatic carboxylic acids (vanillic and syringic acid derivatives, via oxidation) or into ferulic/sinapic acid derivatives (via Perkin or Doebner–Knoevenagel condensation), reactions that are often quantitative.Based on reported average polymer yields exceeding 80% and assuming such quantitative conversion, it can be estimated that approximately 63% of the sustainably available forestry residues processed through OCF would be sufficient to supply the required equivalent of 65 Mt yr^–1^ of TPA-type monomer. Under these assumptions, forestry residues alone could, in principle, meet the required demand.

This estimate likely represents an optimiztic upper bound, as quantitative conversion of complex OCF mixtures may not be fully achievable in practice. Nevertheless, the calculation illustrates a substantial improvement over the earlier TPA substitution strategy. In addition, this approach does not rely on multistep transition-metal-catalyzed transformations and is therefore expected to be more economically feasible and easier to implement at scale. The key challenge, however, will be comprehensive materials characterization to ensure suitability for specific applications. Reported Tg and Tm values alone are not sufficient to assess performance. Properties such as mechanical strength, toughness, gas barrier performance, processability, and long-term durability must also be systematically evaluated.

Several related strategies toward lignin-derived PET analogues have been reported, including approaches based on derivatives of *p*-hydroxybenzoic, vanillic, and syringic acids as well as *p*-coumaric and ferulic acid platforms.^129–135^ Additional examples, not discussed here in detail, include bisphenols obtained from isoeugenol via olefin metathesis,^99^ hydroxyethylated divanillin spiroacetals,^136^ ferulic acid-derived diesters,^137^ bisvanillyl alcohol and vanillic acid dimers,^138–140^ and alicyclic diols obtained via catalytic funneling of lignin oil produced by Cu-PMO-catalyzed RCF.^48^

Within this wide range of strategies for producing lignin-derived PET analogues pathways from hydroxyalkylated *p*-coumaric, ferulic, and sinapic acids appear particularly promising as they rely on high-yielding synthetic routes, are less sensitive to mixture composition as A-B-type monomers, and allow for the potential incorporation of all three lignin-derived core motifs (H, G, S). In many reported cases, Tg values are comparable to PET, whereas Tm values are typically somewhat lower. However, beyond Tg and Tm, comprehensive material properties are rarely reported, leaving open questions regarding mechanical performance, barrier properties, and long-term stability. In parallel, other emerging biobased monomers, such as furan-based diacids, should also be considered when assessing realistic pathways toward large-scale PET alternatives.

### Pathways for PS Analogs

Polystyrene (PS) represents another major class of high-volume aromatic polymers targeted for substitution with lignin-derived building blocks. Produced globally at approximately 22 Mt yr^–1^,^141^ PS is widely used in packaging, disposable food containers, insulation foams, consumer goods housings, and laboratory ware. Its industrial synthesis relies on the monosubstituted vinyl group of styrene, which undergoes radical chain-growth polymerization. As this motif is not present in primary lignin-derived platform molecules, its installation constitutes the key initial step in the development of lignin-derived PS analogues.

The most established route to lignin-derived styrene analogues is the decarboxylation of ferulic or *p*-coumaric acids to yield the corresponding vinylphenols.^142–148^ These cinnamic acid derivatives can be obtained directly from herbaceous lignocellulosic biomass or synthesized via Knoevenagel–Doebner or Perkin condensations of lignin-derived aldehydes such as vanillin, syringaldehyde, and *p*-hydroxybenzaldehyde. Numerous base-promoted and metal-catalyzed protocols have been reported, typically affording yields of 80–90%, making this approach robust and widely applicable. Alternatively, they can be accessed through ethenolysis of propenyl-substituted lignin-derived phenolics.^149,150^ Beyond generating the desired styrenic monomers, this strategy can simultaneously produce valuable coproducts such as propylene or acrylic acid. These transformations rely on ruthenium-catalyzed olefin metathesis and are sensitive to functional groups and impurities commonly present in lignin streams, often requiring careful optimization or purification. Recent advances in catalyst design and impurity-tolerant systems, however, have improved the practicality of this approach.^100,151,152^ A complementary strategy involves carbon-chain extension via Wittig olefination of lignin-derived aldehydes. This transformation has been demonstrated under solvent-free mechanochemical conditions for *p*-hydroxybenzaldehyde, vanillin, and syringaldehyde.^153^ While the classical Wittig reaction suffers from poor atom economy due to stoichiometric phosphine oxide formation, emerging catalytic variants may enhance sustainability, particularly when combined with mechanochemical activation.^154^ Enzymatic decarboxylation routes to vinylphenols have also been reported but are beyond the scope of this Perspective.^155–157^

A defining feature of lignin-derived platforms is the presence of phenolic functionality, which interferes with radical polymerization because unprotected phenols inhibit chain propagation via hydrogen atom transfer. Consequently, protection of the phenolic group is typically required, most commonly through acetylation,^143,158,159^ silylation,^142,158,160^ or methylation.^153^ The resulting chemical space of lignin-derived styrenic monomers explored for PS substitutes is summarized in Figure 5.

**Figure 5:**
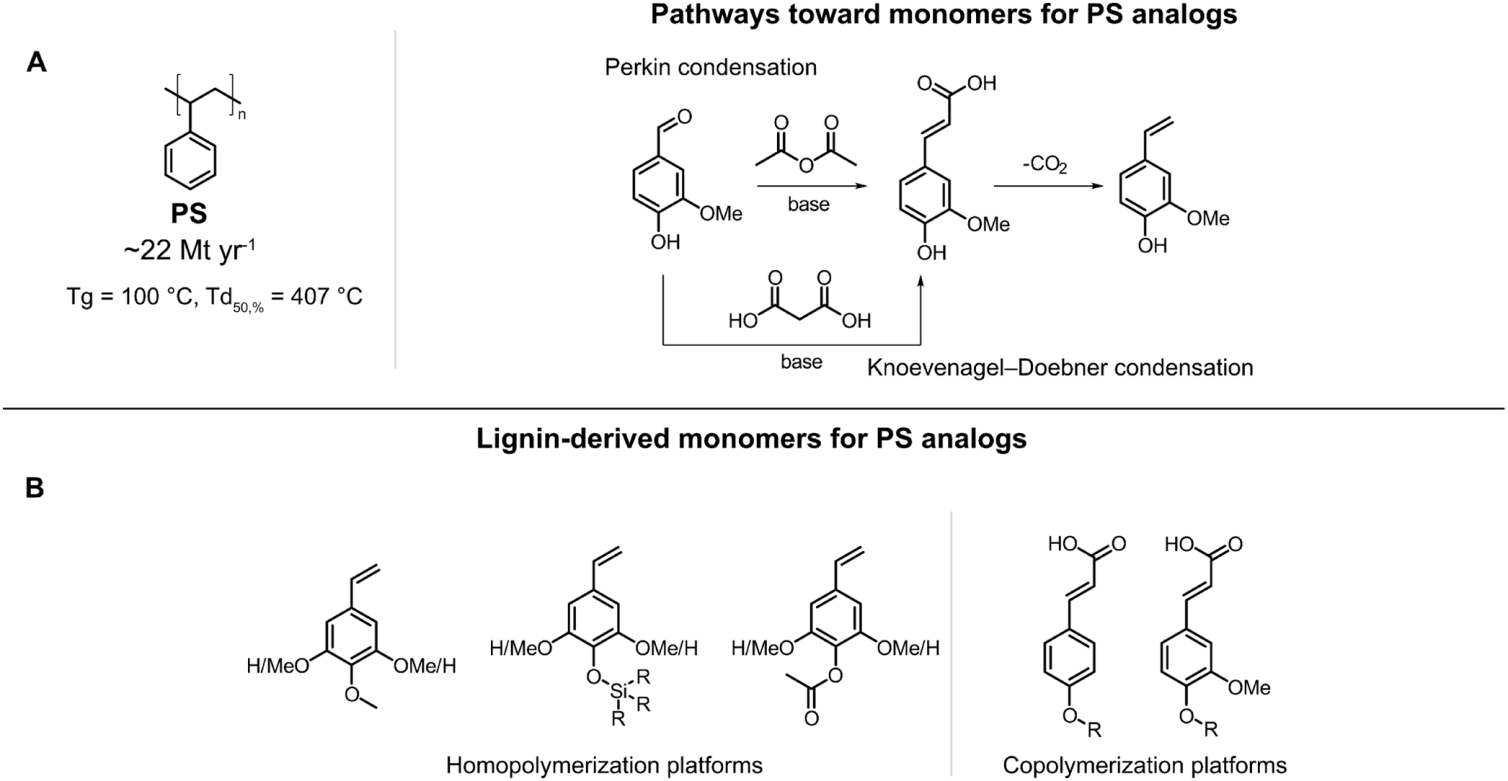
Lignin-derived substitution pathways toward PS. (A) Chemical structure of polystyrene and major synthetic pathways toward styrenic monomers from lignin-derived building blocks. (B) Common structures of lignin-derived monomers suitable for homo- and copolymerization as PS analogues.

Protected vinylphenols generally exhibit polymerization behavior comparable to styrene. Both conventional free-radical polymerization and controlled radical polymerization techniques—such as reversible addition–fragmentation chain-transfer (RAFT) polymerization, atom transfer radical polymerization (ATRP), and nitroxide-mediated polymerization (NMP)—have been successfully applied, affording polymers with Mn ≈ 30–66 kDa and low dispersities under optimized conditions (e.g., Đ ≈ 1.1 in RAFT).^143,147,153,158,160–162^ These results demonstrate that controlled polymerization of lignin-derived styrenic monomers is feasible. However, bulky protecting groups such as triisopropylsilyl can impede reversible chain transfer, resulting in higher dispersities. The thermal stability of protected polymers (Td5% ≈ 360–385 °C) is slightly lower than that of polystyrene. In contrast, Tg depend strongly on substitution pattern and protecting group and cover a wide range. Methylated and silylated derivatives typically exhibit lower Tg (90–110 °C), than acetylated analogues (100–130 °C), while deprotected phenolic polymers show significantly higher Tg (by ∼ 20–50 °C elative to their protected counterparts) due to intermolecular hydrogen bonding. Across substitution patterns, guaiacyl-derived polymers display the lowest Tg, consistent with internal plasticization from asymmetric ortho substitution.

While developing biobased substitutes for high-volume commodity polymers such as PS is an important goal, it is equally compelling to explore the diverse polymer structures and property profiles enabled by phenolic groups. For instance, phenolic functionalities can be leveraged in CO_2_-separating membranes,^147^ redox-active materials,^160^ or as substrates for constructing complex polymer architectures that exploit pendant phenolic groups.^159,163^ One illustrative example is the synthesis of star polymers from epoxidized methoxy vinylphenols, where two consecutive polymerization steps are employed: first, radical copolymerization of styrene or acrylates with epoxy-functionalized vinyl guaiacol, followed by ring-opening polymerization of the pendant epoxides with anhydrides.^164^ Such architectures are of interest for a range of high-end applications, including drug delivery systems and other biomedical uses.

Finally, it is interesting to mention alternative strategies toward PS-like materials from lignin. Cinnamic acid derivatives (*p*-coumaric, ferulic, and sinapic acids) can be viewed as β-substituted styrenes or acrylic acid analogues. Although they are unable to undergo efficient homopolymerization, they can be copolymerized with conventional vinyl monomers under suitable radical polymerization conditions.^165,166^ This strategy enables the direct incorporation of primary lignin-derived building blocks into PS analogs. In addition, the carboxyl functionality of cinnamic acid derivatives provides opportunities to further diversify chemical functionality and modulate the thermal properties of the resulting copolymers.

In terms of matching the production volumes of lignin-derived platforms with those of PS, the choice of depolymerization strategy is critical. Because synthetic routes toward styrenic monomers rely primarily on lignin-derived aldehydes and cinnamic acid derivatives, product streams obtained from RCF are not well suited for this purpose. Based on the estimates shown in Figure 2 OCF represents a more favorable scenario: approximately 22% of a forestry residue streams alone could, in principle, supply the required PS monomer volume, assuming an 80% yield of the corresponding vinylphenols, consistent with literature reports. Although the conversion of lignin-derived aldehydes or cinnamic acids to vinylphenols is synthetically convenient and typically high yielding, these routes remain suboptimal in terms of atom efficiency, as they require removal or installation of carbon atoms in the side chain. Consequently, identifying lignin-derived building blocks that already possess the Ar–C_2_ motif would be highly desirable. One promising example is *p*-hydroxyphenylacetaldehydes, which can be generated selectively through diol-stabilized acidolysis of lignin. Such intermediates could provide a more atom-economical and potentially scalable entry point to styrenic polymer analogues.

Overall, lignin-derived styrenic monomers appear promising from both a supply and synthetic perspective: projected monomer production volumes are compatible with the PS market, and the synthetic routes are generally high yielding and do not rely on transition-metal catalysis. In addition, the presence of phenolic functionality—particularly in derivatives bearing removable protecting groups such as silyl or acetyl—may enable expanded end-of-life strategies, as quantitative deprotection can reveal free phenolic groups that could undergo oxidative cleavage of the benzylic C–C linkage to form benzoquinone-type fragments, analogous to pathways observed during oxidative lignin depolymerization.^167^ However, lignin-derived PS analogues remain far less studied in terms of bulk material performance compared to PET- or BPA-derived systems, making any conclusions regarding their suitability for specific applications largely speculative at this stage. Studies assessing the bulk properties of lignin-based vinylphenols homopolymers and their copolymers with cinnamic acid derivatives are therefore strongly encouraged.

## Conclusions and Future Perspectives

### Lignin-to-Platform Molecules

To date, the only lignin depolymerization strategies that deliver high yields of platform molecules are lignin-first approaches, in which virgin lignocellulosic biomass is treated directly rather than isolating lignin prior to deconstruction. In these processes, lignin is converted in situ to generate monophenolic products. Well-developed RCF methods enable nearly quantitative cleavage of C–O bonds but are unable to cleave C–C bonds, limiting product yields to approximately 20 wt % for softwoods and ∼40 wt % for hardwoods. Oxidative methods can increase yields to ∼50 wt % by enabling partial C–C bond cleavages. Nevertheless, even in the highest yielding methods more than half of the lignin carbon is typically not converted into useful platform molecules. A clear direction for future development therefore lies in high-yielding C–C bond cleavage. One promising strategy is a two-step depolymerization approach, in which lignin is first converted into phenolic monomers and oligomers via efficient C–O bond cleavage, followed by targeted upgrading of the remaining oligomeric fraction. This strategy may be advantageous because C–O and C–C bond activation likely require different optimal reaction environments, and applying a single set of conditions could compromise overall product yields. Early examples of such tandem approaches already demonstrate access not only to additional amounts of monomers, but also to products with alternative structures, e.g., benzoquinone derivatives.^167–169^

### Lignin-Platforms-to-Monomers

The upgrading of primary lignin-derived platforms into precursors for commercial products, such as monomers for polymer synthesis, must reflect the fundamental differences between lignin- and petroleum-derived streams. Petroleum refineries operate on simple, nonoxygenated platform molecules such as ethylene, propylene, benzene, and toluene, which are progressively functionalized to build molecular complexity. In contrast, lignin depolymerization directly yields highly functionalized aromatic compounds. Efficient upgrading strategies should therefore prioritize the utilization of the inherent functionality present in lignin-derived molecules before pursuing extensive defunctionalization. In this sense, lignin valorization follows a trajectory of selective simplification, rather than the complexity-building paradigm typical of petrochemical refining. The chemical space accessible from primary lignin platforms provides numerous multifunctional molecules that can potentially be used directly as monomers. Ferulic and *p*-coumaric acids are illustrative examples. These compounds occur in lignin depolymerization streams obtained from agricultural residues or can be readily accessed from aldehydes (vanillin, *p*-hydroxyphenyl aldehyde) produced by OCF of woody biomass. As A-B-type monomers, they can be directly polymerized into polyesters, with PET-like materials representing a primary target. Such direct utilization pathways should be explored first, evaluating the performance of the resulting materials which will govern their most suitable applications. At the same time, some applications require monomers with reduced functionality. For these cases, selective defunctionalization strategies—including demethylation, demethoxylation, dealkylation, and controlled hydrogenation—should be further developed under milder and more efficient conditions.

Another key challenge is the compositional complexity of lignin-derived product streams. Unlike petroleum hydrocarbons, lignin-derived aromatics are highly oxygenated, giving them high boiling points and narrowly spaced volatilities that make separation by distillation difficult. Consequently, strategies that tolerate or directly utilize mixtures should be prioritized. For example, mixtures containing guaiacyl and syringyl derivatives may be suitable for thermoset resins or amorphous thermoplastics, whereas streams with lower compositional complexity—such as those derived from softwoods—may be preferable for applications requiring high structural regularity, such as polyester fibers. Finally, catalytic funneling strategies that convert complex mixtures into single products remain highly important for extracting value from otherwise inseparable streams. However, such approaches may be most effective when applied selectively after opportunities for direct utilization have been thoroughly explored.

### Lignin-Derived Monomers-to-Materials

At an early stage of integrating lignin-derived platforms into materials value chains, targeting high-volume commercial polymers represents a pragmatic strategy and remains the most common approach to date. In this context, it is important to assess whether the potential production volumes of lignin-derived monomers can realistically match market demand. Very rough estimates performed in this study suggest that forestry residues alone could, in principle, supply lignin-derived platforms for major aromatic polymers, including PET, BPA-based materials, and PS. Under optimiztic assumptions based on laboratory-scale yields, OCF could provide sufficient monomer equivalents if approximately 63% of forestry residues were directed toward PET analogues, 22% toward PS analogues, and 13% toward BPA-based materials. Although these estimates represent best-case scenarios, they illustrate potential of lignin streams meaningful contribution to materials production. However, the viability of lignin-derived polymers ultimately depends not only on feedstock availability but also on material performance and economic competitiveness. At present, key properties such as mechanical strength, durability, rheology, and barrier performance remain insufficiently characterized, limiting meaningful benchmarking against commercial materials. Nevertheless, several general trends can already be identified. The structural diversity of lignin-derived monomers allows access to polymers with a broad Tg range. Rigid aromatic cores and polar functional groups (e.g., carboxyl groups) tend to increase Tg, whereas flexible aliphatic side chains and methoxy substituents typically reduce it. The high functionality of lignin-derived platforms—including phenolic groups, and reactive side-chain motifs—enables diverse polymerization and functionalization strategies. This functionality can facilitate the direct incorporation of useful chemical features into polymer backbones, potentially reducing the need for additives. On the other hand, the highly oxygenated nature of lignin-derived monomers can lead to reduced thermal stability and increased water uptake, and methoxy substitution often suppresses crystallization and may limit the formation of highly ordered materials.

These trade-offs point toward a fundamental shift in design philosophy. Rather than replicating existing polymer structures from lignin-derived feedstocks, polymer design should be application-driven: the performance requirements of a given use case should be defined first, and then met through suitable combinations of lignin-derived building blocks, guided by established structure–property relationships. In practice, this means that applications currently served by a single commodity polymer might in the future be addressed by several different lignin-derived materials, each tailored to the specific performance requirements of its application.

### Future Biorefineries, Larger Picture

Finally, when considering the long-term development of lignin-based biorefineries in a broader context, two conceptual directions can be envisioned: feedstock- and product-agnostic systems, and feedstock- or product-specific processing models. Each approach offers distinct advantages and limitations. Agnostic systems aim to process a wide range of biomass feedstocks into diverse products using a limited set of robust primary processing steps combined with flexible downstream upgrading. In this context, tandem strategies that integrate reductive and oxidative depolymerization may be particularly promising. For example, initial reductive cleavage of C–O bonds can provide stable lignin-derived intermediates and partially defunctionalized oligomeric substrates, which can subsequently undergo selective oxidative C–C bond cleavage. This stepwise approach will also enable a broader spectrum of aromatic building blocks including oxidized and reduced products. Such agnostic strategies could simplify biorefinery design by relying on a limited number of core processes that can be optimized for efficiency and scalability.

In contrast, feedstock- and product-specific processing models aim to maximize value extraction by tailoring processing conditions to the composition of the biomass and the requirements of the target products. Advances in analytical techniques and machine-learning-based modeling may enable such approaches. Predictive models that relate feedstock composition, molecular structure, reaction conditions, and material performance could guide the selection of catalytic depolymerization and upgrading pathways. In this framework, biomass streams would be continuously characterized and directed toward the most suitable transformation routes, while upstream fractionation conditions are tuned to generate intermediates optimized for downstream applications such as polymer synthesis. While this strategy could enable more efficient utilization of biomass resources, it would require more complex modular and adaptable processing infrastructure.

Overall, the quantitative analysis presented in this Perspective should be regarded as a rough first-order feasibility assessment rather than a reliable production forecast. The estimates, derived from lab-scale protocols under optimiztic assumptions, indicate that sustainably available lignocellulosic residues could in principle supply aromatic monomers at scales broadly comparable to current petrochemical markets. Whether this potential is practically realizable remains an open question, as the estimates do not account for the collection and transportation of lignocellulosic residues, the costs of product isolation and purification, or the substantial challenges of process scale-up. What can be concluded with greater confidence is that lignin-to-materials pathways are sufficiently promising to be seriously considered as contributors to at least part of future materials production. More realistic feasibility estimates will require research spanning biomass logistics, process scale-up, catalyst stability under industrially relevant conditions, and techno-economic and life-cycle assessments.

Another key remaining challenge is to establish clear relationships between monomer structure, and application performance through systematic evaluation of materials in the context of targeted applications, rather than direct comparison with existing petroleum-based counterparts. New materials that capitalize on the inherent functionality of lignin-derived building blocks should therefore be designed and assessed, increasingly supported by the predictive capabilities of modern machine-learning methods. Importantly, matching or surpassing the performance of established commercial materials should be regarded as a long-term objective rather than an immediate expectation, since current polymers achieved their present performance only through decades of iterative development by scientists and engineers across multiple disciplines.
